# BAFF Blockade Attenuates Inflammatory Responses and Intestinal Barrier Dysfunction in a Murine Endotoxemia Model

**DOI:** 10.3389/fimmu.2020.570920

**Published:** 2020-11-26

**Authors:** Runze Quan, Chaoyue Chen, Wei Yan, Ying Zhang, Xi Zhao, Yu Fu

**Affiliations:** ^1^Department of Gastroenterology, Union Hospital, Tongji Medical College, Huazhong University of Science and Technology, Wuhan, China; ^2^Department of Gastroenterology, Tongji Hospital, Tongji Medical College, Huazhong University of Science and Technology, Wuhan, China

**Keywords:** B cell activating factor, endotoxemia, inflammation, tight junction, intestinal mucosal barrier

## Abstract

B cell-activating factor (BAFF) production is increased in septic patients. However, the specific role of BAFF in sepsis remains unknown. This study was designed to investigate the expression and function of BAFF in an experimental endotoxemia model and to identify the potential mechanisms. We established an endotoxemia mouse (6–8 weeks, 20–22 g) model by administering 30 mg/kg lipopolysaccharide (LPS). BAFF levels in the circulating system and organ tissues were measured 4 and 8 h after LPS injection. Survival rates in the endotoxemia mice were monitored for 72 h after BAFF blockade. The effects of BAFF blockade on systemic and local inflammation, organ injuries, and intestinal barrier function were also evaluated 4 h after LPS treatment. BAFF production was systemically and locally elevated after LPS challenge. BAFF blockade improved the survival rate, systemic inflammation, and multi-organ injuries. Moreover, BAFF blockade attenuated both intestinal inflammation and impaired intestinal permeability. BAFF blockade upregulated ZO-1 and occludin protein levels *via* the NF-κB/MLCK/MLC signaling pathway. These results suggested that BAFF blockade protects against lethal endotoxemia at least partially by alleviating inflammation, multi-organ injuries, and improving intestinal barrier function and provides a novel focus for further research on sepsis and experimental evidence for clinical therapy.

## Introduction

Sepsis is a life-threatening multi-organ dysfunction that results from dysregulated host immune response to microorganism infection (1). Despite advances in medical treatment, the mortality rate of patients with sepsis remains alarmingly high (2). Systemic and local immunological dissonance triggered by microbial infection results in an uncontrolled inflammatory response and subsequent multi-organ dysfunction (3). The intestinal tract is an important effector organ for sepsis and is considered the motor of critical illness (4).

Intestinal barrier impairment plays a vital role in the development of systemic sepsis (4). Intestinal hyperpermeability is associated with alterations in tight junctions (TJ) between intestinal epithelial cells caused by intestinal hypoperfusion and hypoxia (5). Increased intestinal permeability allows the translocation of enteric flora and endotoxins into the blood and can lead to gut-derived infection and enhanced systemic inflammatory response (6). Therefore, the intestinal barrier could be a potential target for sepsis therapy.

B cell-activating factor (BAFF), a member of the tumor necrosis factor (TNF) superfamily, is predominantly expressed by myeloid cells but is also produced by other cells, including epithelial cells (7, 8). BAFF binds to three different receptors: BAFF-receptor, B cell maturation antigen, and transmembrane activator and calcium-modulating and cyclophilin ligand interactor (8). BAFF binding to its receptors activates downstream signaling pathways that contribute to B cell survival and maturation and the regulation of B cell and T cell function (9). Mounting evidence has revealed that BAFF is associated with several inflammatory diseases, including systemic lupus erythematosus, rheumatoid arthritis, and inflammatory bowel diseases (8). Recently, Dajana et al. found that BAFF was highly expressed in the serum of patients with sepsis (10). However, the specific role of BAFF in the immunopathogenesis of sepsis remains largely unknown. Here, we sought to investigate the expression and function of BAFF in a lipopolysaccharide (LPS)-induced mouse endotoxemia model, and to identify the underlying mechanisms.

## Materials and Methods

### Animals

Male C57BL/6 mice (6–8 weeks, 20–22 g) were purchased from Beijing Vital River Laboratory Animal Technologies Co. Ltd. All mice were fed under specific pathogen free conditions in the animal facility of Tongji Medical College with free access to sterile water and food. The mice were housed for one week at room temperature (25°C) with 12 h light/dark cycles for climatization before commencement of experiments. Animal experiments were approved by the Animal Management and Use Committee of Huazhong University of Science and Technology (no. S2366).

### LPS-Induced Endotoxemia Model and Treatments

LPS from *Escherichia coli* O55:B5 (Sigma Aldrich) was dissolved in sterile phosphate-buffered saline (PBS). Mice were intraperitoneally injected with LPS (30 mg/kg) or equivolumetric PBS. For BAFF blockade during experimental endotoxemia, 50 µg of mouse BAFF monoclonal antibody (Sandy-2, Adipogen Life Sciences) was administered intraperitoneally 30 minutes after LPS challenge. As a control, mouse immunoglobulin G1 (IgG1) isotype control antibody (Adipogen Life Sciences) was used. BAFF expression in mice with endotoxemia was explored by randomly dividing all mice into control (n = 10), LPS 4 h (n = 10), LPS 8 h (n = 10), and LPS + anti-BAFF 4 h (n = 10) groups. For other experiments, mice were randomly allocated into control (n = 10), LPS 4 h (LPS, n = 10), and LPS + anti-BAFF 4 h (LPS + anti-BAFF, n = 10) groups. Mice were euthanatized and organ tissues were removed at the indicated time points after LPS injection. To investigate the effect of BAFF antibody intervention on LPS-induced mortality, survival was monitored every 12 h for 3 days.

### Histopathological Examinations

Small segments of distal ileum were obtained and fixed with 4% paraformaldehyde for 24 h at room temperature. Collected tissue samples were then embedded in paraffin, serially sectioned at 4-µm thickness and stained with hematoxylin and eosin (H&E) for morphological analysis. Chiu’s score was used to evaluate intestinal injuries (11).

### Enzyme-Linked Immunosorbent Assay (ELISA)

BAFF concentrations in serum or tissue homogenates were detected using the mouse BAFF quantikine ELISA kit (R&D systems) following the manufacturer’s instructions. Concentrations of interleukin 1β (IL-1β), interleukin 6 (IL-6), interleukin 10 (IL-10), tumor necrosis factor α (TNF-α) and myeloperoxidase (MPO) were also measured using ELISA kits (Bioswamp).

### Serum Biochemical Markers for Multi-Organ Damage

To determine the effect of BAFF antibody on multiple organ injury, blood alanine aminotransferase (ALT), aspartate aminotransferase (AST), creatinine (Cr), and lactate dehydrogenase (LDH) were assessed using an Automatic Biochemical Analyzer (Rayto Life and Analytical Sciences Co., Ltd).

### Evaluation of Intestinal Barrier Damage

Serum was harvested from mice 4 h after LPS treatment, and the levels of diamine oxidase (DAO) and D-lactate were examined using ELISA kits (Bioswamp) following the manufacturer’s instructions. Two hours after LPS administration, mice were given 750 mg/kg fluorescein isothiocyanate (FITC)-dextran 4 kDa (FD-4) by oral gavage and blood samples were collected 2 h after gavage. Then, the serum was separated and the concentration of FD-4 in the serum was measured using a fluorescence spectrophotometer (Thermo Fisher Scientific) at excitation and emission wavelengths of 490 nm and 520 nm, respectively.

### Quantitative Real-Time Polymerase Chain Reaction (qPCR)

Primary intestinal epithelial cells were isolated as described previously (12). Total RNA from intestinal tissues or isolated primary cells were extracted using Trizol (Invitrogen) methods and reverse transcribed into cDNA using the PrimeScript™ RT reagent Kit (Takara) following the manufacturer’s instructions. Quantitative PCR analyses were performed on a LightCycler^®^ 480 System (Roche) using the SYBR Green PCR Master Mix (Takara, Japan). The relative amount of RNA for each gene was normalized against β-actin and analyzed using the 2^–ΔΔCt^ method. Amplification procedures were: pre-denaturation at 95°C for 10 min, and 40 cycles of denaturation at 95°C for 30 s, annealing at 60°C for 1 min, and extension at 72°C for 30 s. Primers used in this experiment were: Mouse *IL-1β:* 5’-GCAACTGTTCCTGAACTCAACT-3’ and 5’-ATCTTTTGGGGTCCGTCAACT-3’. Mouse *IL-6:* 5’- GAGAAAAGAGTTGTGCAATG-3’ and 5’-ATTTTCAATAGGCAAATTTC-3’. Mouse *IL-10:* 5’-GCTCTTACTGACTGGCATGAG-3’ and 5’-CGCAGCTCTAGGAGCATGTG-3’. Mouse *TNF-α:* 5’-TACTGAACTTCGGGGTGATTGGTCC-3’ and 5’-CAGCCTTGTCCCTTGAAGAGAACC-3’. Mouse *β-actin:* 5’- GGCTGTATTCCCCTCCATCG-3’ and 5’-CCAGTTGGTAACAATGCCATGT-3’.

### Western Blot

Total protein was extracted from intestinal tissues using radioimmunoprecipitation assay (RIPA) buffer (Beyotime Biotechnology) containing a protease and phosphatase inhibitor Cocktail (MedChemExpress). Proteins were quantified using a bicinchoninic acid (BCA) protein assay kit (Thermo Fisher Scientific). Protein lysates were separated by 10 or 7.5% sodium dodecyl sulfate polyacrylamide gel electrophoresis (SDS-PAGE) and transferred onto polyvinylidene difluoride membranes. Membranes were blocked with blocking buffer composed of 5% bovine serum albumin (Antgene Biotechnology) in Tris-buffered saline with 0.1% Tween-20 for 1 h at room temperature. Membranes were then incubated with primary antibodies and horse radish peroxidase (HRP)-conjugated secondary antibodies (Antgene Biotechnology). Proteins were visualized using an electrochemical luminescence (ECL) kit (Thermo Fisher Scientific) and chemiluminescence imaging system (Bio-Rad). The following primary antibodies were used: anti-zonula occludens (ZO)-1 (Thermo Fisher Scientific), anti-occludin (ABclonal Technology), myosin light chain kinase (MLCK) (Immunoway), myosin light chain (MLC) (Cell Signaling Technology), phospho-MLC (p-MLC) (Cell Signaling Technology), nuclear factor kappa B (NF-κB) p65 (ABclonal Technology), p-p65 (ABclonal Technology), IκB-α (ABclonal Technology), p-IκB-α (ABclonal Technology), and glyceraldehyde-phosphate dehydrogenase (GAPDH) (Antgene Biotechnology).

### Statistical Analysis

Data are shown as mean ± S.D. GraphPad Prism 7.0 software was applied for statistical analysis and plotting. Survival analysis was performed using the Kaplan-Meier method. Differences among groups were compared using One-way-analysis of variance (ANOVA) or Mann-Whitney U test. P value < 0.05 was considered statistically significant.

## Results

### BAFF Expression Is Systemically and Locally Upregulated in Endotoxemia Mice

**Figure 1:**
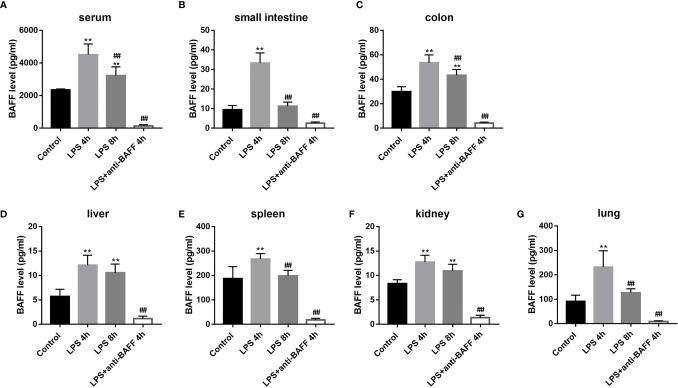
Systemic and local BAFF production in LPS-induced septic mice. Septic mice (n=10 for each group) were intraperitoneally injected with 50ug anti-BAFF antibody or control antibody. Blood samples and organs were collected at the indicated time points. Samples were assayed for BAFF concentrations by ELISA. **(A–G)** BAFF concentrations in the serum, small intestine, colon, liver, spleen, kidney, and lung tissues, respectively. *and** indicate P<0.05 and P<0.01, respectively compared with control group; ^##^ indicates P<0.01 compared with LPS 4h group.

BAFF production is enhanced in the serum of patients with sepsis (10). Here, we analyzed BAFF expression in the well-established experimental mouse endotoxemia model using ELISA. In the LPS-induced mice endotoxemia model, BAFF levels are significantly upregulated in the serum, small intestine, colon, liver, spleen, kidney, and lung tissues 4 h and 8 h after LPS administration (**Figure 1**). Although there were no significant differences in the hepatic and renal tissues between LPS 4 h and LPS 8 h groups, BAFF levels in the serum and other organ tissues were higher 4 h after LPS challenge. Therefore, mice in LPS 4 h group were chosen for subsequent experiments. In the LPS + anti-BAFF group, BAFF concentrations were extremely low indicating that BAFF was successfully neutralized.

### BAFF Blockade Improves Survival and Organ Functions in Endotoxemia Mice

**Figure 2:**
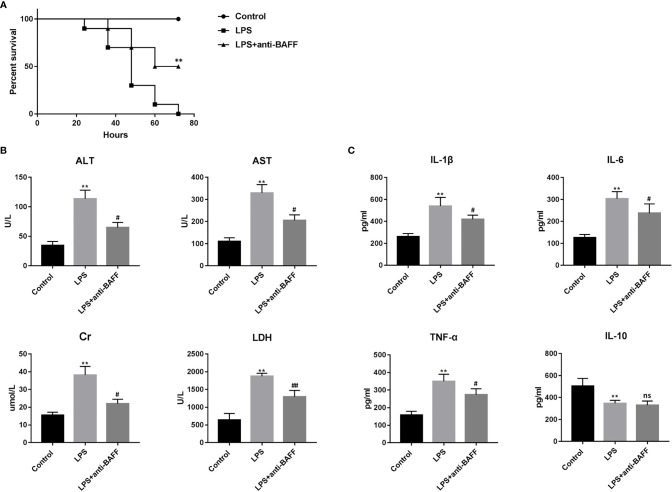
Effects of BAFF blockade on survival, multiple organ dysfunction and systemic inflammatory response of LPS-induced septic mice. Septic mice (n=10 for each group) were intraperitoneally injected with 50ug anti-BAFF antibody or control antibody. Mice were euthanatized and blood samples were collected 4 h after LPS challenge. **(A)** Survival rate of septic mice in different groups. ** indicates P<0.01 compared with LPS group. **(B)** Levels of serological markers for organ injury, including ALT, AST, Cr and LDH. **P<0.01 compared with control group; ^#^ and ^##^ indicates P<0.05 and P<0.01, respectively compared with LPS group. **(C)** Levels of inflammatory cytokines in the serum, including IL-1β, IL-6, TNF-α and IL-10. **P<0.01 compared with control group; # indicate P<0.05 compared with LPS group.

To investigate the role of BAFF in the experimental endotoxemia model, we observed the effects of BAFF blockade on LPS-induced mortality (**Figure 2A**). Significant survival rate improvement was observed in endotoxemia mice treated with the BAFF antibody.

Since multi-organ dysfunction contributes to the lethality of sepsis, we then detected several serum clinical biomarkers as indicators of organ function. Serum ALT and AST levels, markers of hepatocellular injury, creatinine, a marker of renal dysfunction, and LDH, a marker of general cellular injury, were significantly elevated after LPS treatment (**Figure 2B**). Intraperitoneal BAFF antibody administration dramatically reversed the elevation of these biomarkers, indicating that BAFF blockade protects against LPS-induced multiple organ injury.

### BAFF Blockade Downregulates Inflammatory Cytokine Serum Levels

Excessive inflammatory response is associated with multiple organ failure and poor outcome in sepsis, and is characterized by enhanced expression of inflammatory factors including IL-1β, IL-6, and TNF-α (13, 14). We examined serum concentrations of IL-1β, IL-6, TNF-α, and IL-10 to investigate the effect of BAFF antibody on systemic inflammation in endotoxemia. Our results show that serum IL-1β, IL-6, and TNF-α levels were significantly increased after LPS challenge, while serum IL-10 levels decreased (**Figure 2C**). BAFF antibody treatment resulted in a significant reduction in the serum concentrations of IL-1β, IL-6, and TNF-α but not IL-10 after LPS injection. These results suggest that BAFF blockade attenuates the systemic inflammatory response in endotoxemia.

### BAFF Blockade Alleviates LPS-Induced Intestinal Mucosal Injury

**Figure 3:**
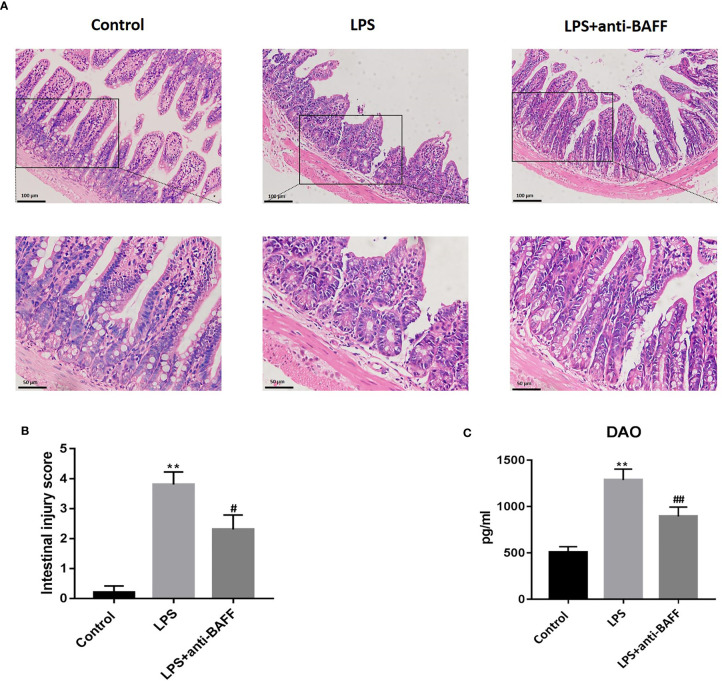
Effects of BAFF blockade on intestinal mucosal injury. Septic mice (n=10 for each group) were intraperitoneally injected with 50ug anti-BAFF antibody or control antibody. Mice were euthanatized and samples were collected 4 h after LPS challenge. **(A)** Pathological photomicrographs of ileum sections with H&E staining of mice in different groups. Magnification x 200 or 400. **(B)** Chiu’s score of intestinal tissue. **(C)** Serum levels of DAO in different groups. **P<0.01 compared with control group; ^#^ and ^##^ indicate P<0.05 and P<0.01, respectively compared with LPS group.

Intestinal damage and barrier dysfunction are closely related to sepsis (15). Intestinal mucosal morphology was observed by H&E staining under an optical microscope. Intact ileum mucosa, with neatly arranged villi and glands, was observed in the control group, while mice treated with LPS showed severe mucosal damage characterized by denuded villi with dilated capillaries, exposed lamina propria, and inflammatory cell infiltration (**Figure 3A**). The LPS-induced intestinal mucosal injuries were significantly improved by BAFF blockade. Morphological injuries were evaluated by Chiu’s score (**Figure 3B**). DAO is normally expressed by ciliated epithelial cells in the small intestinal mucosa and, following massive epithelial damage, is released into systemic circulation. We examined serum DAO levels by ELISA. DAO concentrations were remarkably increased after LPS challenge and this elevation was reversed following BAFF antibody administration, suggesting that anti-BAFF treatment played a protective role against LPS-induced intestinal epithelial injuries (**Figure 3C**).

### BAFF Blockade Attenuates Intestinal Inflammation and Improves Intestinal Permeability

**Figure 4:**
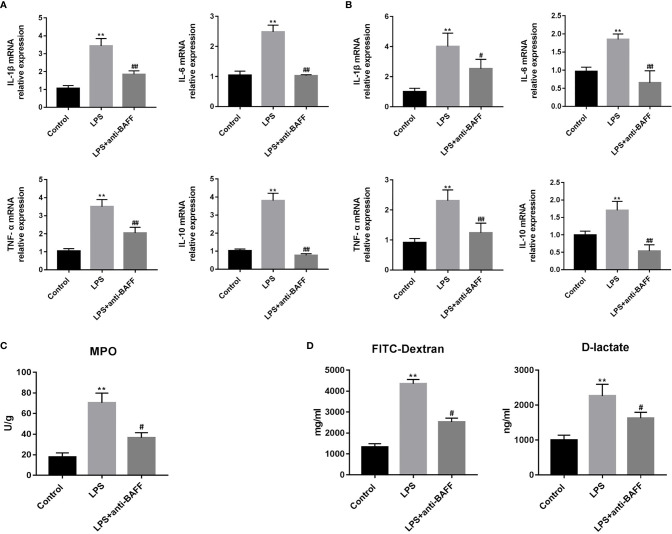
Effects of BAFF blockade on intestinal inflammation and permeability. Septic mice (n=10 for each group) were intraperitoneally injected with 50ug anti-BAFF antibody or control antibody. Mice were euthanatized and samples were collected 4 h after LPS challenge. **(A)** The mRNA expression of inflammatory cytokines in the ileum tissues, including IL-1β, IL-6, TNF-α and IL-10. **(B)** The mRNA expression of inflammatory cytokines of primary intestinal epithelial cells, including IL-1β, IL-6, TNF-α and IL-10. **(C)** MPO concentrations in the ileum tissues of different groups. **(D)** The levels of FITC-dextran 4kDa and D-lactate in the serum of different groups, which reflects the intestinal permeability. **P<0.01 compared with control group; ^#^ and ^##^ indicate P<0.05 and P<0.01, respectively compared with LPS group.

Excessive intestinal inflammation and cytokine storm lead to impaired intestinal permeability in sepsis. We measured the IL-1β, IL-6, TNF-α, and IL-10 mRNA in ileum tissues (**Figure 4A**). We found that IL-1β, IL-6, TNF-α, and IL-10 expression was increased in the LPS group, and that BAFF blockade significantly lowered their expression. Primary small intestinal epithelial cells play a vital role in regulation of inflammatory balance and maintaining of intestinal barrier. Primary small intestinal epithelial cells were isolated and we determined whether BAFF blockade had an effect on their expression of cytokines. LPS-induced production of IL-1β, IL-6, TNF-α, and IL-10 in primary intestinal epithelial cells was reduced by anti-BAFF administration (**Figure 4B**). MPO is enriched in neutrophils and well accepted to be an indicator of inflammation in sepsis (16). To determine the degree of local neutrophil infiltration, MPO activity was examined in ileum tissues by ELISA. LPS injection significantly enhances production of intestinal MPO and MPO concentrations are reduced in the LPS + anti-BAFF group (**Figure 4C**).

In this study, intestinal barrier function was indirectly evaluated by intestinal permeability. Intestinal permeability was determined by serum FD-4 and D-lactate concentrations. D-lactate is a metabolite of intestinal flora that cannot pass intact intestinal barriers. However, FD-4 and D-lactate can penetrate into the bloodstream through injured or compromised intestines. LPS treatment resulted in a significant increase in serum FD-4 and D-lactate levels, while these increases were reversed when BAFF was blocked by the anti-BAFF antibody (**Figure 4D**). These results show that BAFF blockade can alleviate LPS-induced intestinal barrier damage in mice with endotoxemia.

### BAFF Blockade Enhances ZO-1 and Occludin Expression *via* NF-κB/MLCK/MLC Signaling

**Figure 5:**
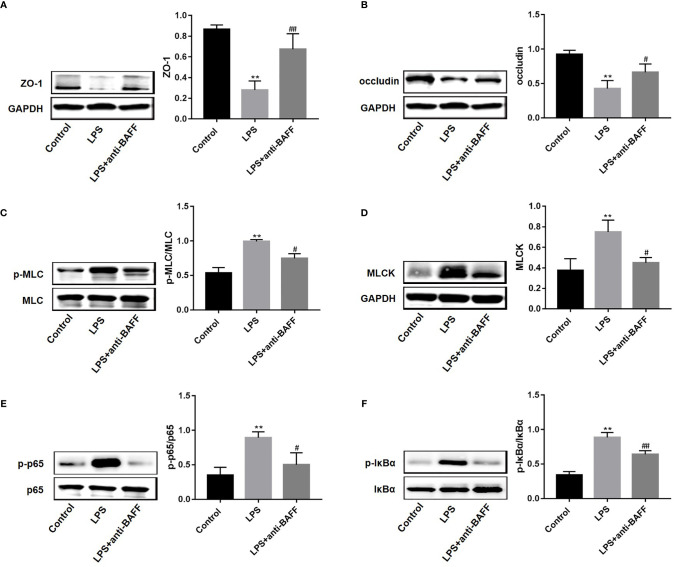
BAFF blockade increases the protein levels of ZO-1 and occludin *via* NF-κB/MLCK/MLC signaling. Mice were euthanatized and samples were collected 4 h after LPS challenge. The expression of proteins was determined by western blot (n=3 for each group). Representative blots were shown and densitometric quantification data were expressed as the intensity ratio of target protein to GAPDH or corresponding controls. **(A)** ZO-1; **(B)** occludin; **(C)** p-MLC/MLC; **(D)** MLCK; **(E)** p-p65/p65; **(F)** p-IκB-α/IκB-α. *and** indicate P<0.05 and P<0.01, respectively compared with control group; ^#^ and ^##^ indicate P<0.05 and P<0.01, respectively compared with LPS group.

TJ proteins participate in the maintenance of intestinal mucosal barrier integrity (15). To investigate whether BAFF antibody regulates TJ proteins to improve intestinal permeability, ZO-1 and occludin expression in ileum tissues was measured by western blot. ZO-1 and occludin protein levels were reduced after LPS challenge, and anti-BAFF treatment rescued their expression levels (**Figures 5A, B**). Emerging evidence shows that activation of MLCK/MLC signaling mediates intestinal barrier damage through the regulation of TJ proteins (17). Therefore, we next examined the levels of MLCK protein and MLC phosphorylation (**Figures 5C, D**). Activation of the MLCK/MLC signaling pathway was markedly induced in mice with endotoxemia, and anti-BAFF treatment inhibited this activation. Furthermore, NF-κB signaling mediates MLCK/MLC pathway activation \ (17, 18). To confirm whether the NF-κB signaling pathway is involved in BAFF-mediated regulation of TJ proteins, NF-κB signaling activation status was tested by western blot. The levels of p65 and IκBα phosphorylation in the LPS group were significantly increased (**Figures 5E, F**). However, anti-BAFF intervention remarkably reduced p-p65 and p-IκBα levels. Collectively, these results suggest that anti-BAFF treatment enhances ZO-1 and occludin expression *via* NF-κB/MLCK/MLC signaling pathway inhibition.

## Discussion

Accumulating evidence shows that BAFF is a crucial regulatory mediator in several inflammatory diseases (8). For example, BAFF expression is enhanced in patients with systemic lupus erythematosus or rheumatoid arthritis, and is positively associated with disease activity (19, 20). Elevated BAFF levels were also found in the gastric mucosa of Helicobacter (Hp) positive patients with chronic gastritis (21). Additionally, BAFF production is augmented in patients with inflammatory bowel diseases. Zhang et al. found that BAFF levels were observably higher in the serum, colon tissues, and stools of patients with ulcerative colitis or Crohn’s disease than in healthy individuals (22). Recently, BAFF concentrations were reported to be elevated in the serum of septic patients and positively correlated with IgM, C3 and C4 complement component serum levels (10). However, BAFF levels were not associated with sepsis disease severity, as reflected by Acute Physiology and Chronic Health Evaluation II and Sequential Organ Failure Assessment scores (10). In this study, we found that BAFF expression was increased in the systemic circulation and in multiple organ tissues of LPS-induced endotoxemia mice, which is consistent with the previous results reported in human samples.

Elevated BAFF levels in the experimental endotoxemia model suggest that BAFF may be involved in the pathogenesis of sepsis. BAFF has been shown to have pro-inflammatory properties in several experimental models of inflammatory diseases. In mice with collagen-induced arthritis, intra-articular BAFF gene silencing significantly ameliorates arthritis development through targeting T helper (Th) 17 cell generation and inhibiting pro-inflammatory cytokine production (23). In a mouse model of primary Sjögren’s syndrome (pSS), BAFF overexpression increased local lymphocytic infiltration in targeted tissues and promoted B cell differentiation (24). Moreover, BAFF antagonist administration suppressed experimental encephalomyelitis and inhibited Th1-related cytokine expression (25). Therefore, to investigate the exact function of BAFF, we used an anti-BAFF antibody to block BAFF in this model. As expected, an improved survival rate was observed after BAFF blockade, and was associated with remission of multiple organ dysfunction as reflected by decreased ALT, AST, Cr, and LDH serum levels.

BAFF promotes the production of several inflammatory mediators in Hp-related chronic gastritis, including IL-1β, IL-6, IL-23, and TGF-β (21) (26). In sepsis, cytokine storm contributes to multi-organ dysfunction and death. Previous studies have clearly indicated the importance of IL-1β, IL-6, IL-10, and TNF-α in sepsis (4, 13, 27, 28). Here, our results show that blockade of BAFF activity suppresses systemic and local inflammation, characterized by decreased serum and intestinal IL-1β, IL-6, and TNF-α levels, in LPS-induced endotoxemia. We also show that the expression of these cytokines was also reduced in primary intestinal epithelial cells following anti-BAFF administration. However, BAFF blockade only altered IL-10 expression in the intestine and primary intestinal epithelial cells, and not in the systemic circulation.

The gastrointestinal tract is an important target organ in sepsis. Under septic conditions, local inflammation, ischemia, and hypoxia damage gastrointestinal tract and intestinal barrier function (15). The translocation of intestinal bacteria and toxins into the blood stream leads to the deterioration of sepsis and distant organ damage (29). In this study, LPS-induced enteric pathologic injuries were observed, and BAFF blockade significantly alleviated these changes. The intestinal mucosal barrier function is largely maintained by physical and functional immunological barriers (30). The physical barrier mainly consists of cellular constituents, including epithelial cells and TJ proteins, while the functional immunological barrier includes various cytokines, immune molecules, and antibacterial peptides (30). Here, BAFF blockade reversed the observed decrease in ZO-1 and occludin protein levels, suggesting that the intestinal barrier was physically protected by BAFF antibody treatment. Moreover, BAFF blockade also reduced the expression of IL-1β, IL-6, and TNF-α pro-inflammatory cytokines, and MPO activity was suppressed, indicating that BAFF blockade also protected against functional immunological barrier loss.

Activation of MLCK/MLC signaling mediates the opening of TJs (17, 31). In this study, LPS increased MLCK and p-MLC protein expression and BAFF antibody treatment attenuated these changes. Collectively, these results suggest that BAFF blockade rescues TJ proteins *via* MLCK/MLC signaling inhibition. Additionally, BAFF can activate the canonical and non-canonical NF-κB pathways in myeloid and lymphoid cells (26, 32, 33). Previous studies have shown that NF-κB signaling triggers MLCK protein expression (17, 18). We demonstrated that NF-κB signaling is involved in MLCK/MLC pathway regulation, as reflected by p65 and IκB-α phosphorylation levels. Collectively, BAFF blockade protects intestinal barrier function *via* NF-κB/MLCK/MLC signaling pathway regulating.

To the best of our knowledge, this is the first study examining the role of BAFF in experimental sepsis. We elucidated that BAFF is potentially involved in the development of LPS-induced endotoxemia. We reached the following conclusions: (1) BAFF is overexpressed in the serum and in multiple organ tissues of mice with endotoxemia; (2) BAFF blockade improves survival rate and multi-organ dysfunction, and alleviates systemic and intestinal inflammatory response; and (3) BAFF blockade improves intestinal barrier function *via* upregulating the expression of ZO-1 and occludin TJ proteins. However, our study does have still some limitations. Firstly, we were not able to recruit patients with sepsis in this study. Further validation of BAFF expression and function in patients would make our support and confirm our results. Secondly, we only investigated the role of BAFF in a murine model of endotoxemia. The effect of BAFF in other sepsis models, including the cecal ligation and puncture models, still needs to be explored. Additionally, we focused more on intestinal barrier function in the study, and the effect of BAFF on dysfunction in other organs should be elucidated in future studies.

We revealed that BAFF plays a detrimental role in a murine model of endotoxemia. Blockade of BAFF protects against lethal endotoxemia in part by alleviating inflammatory response and intestinal barrier dysfunction. Thereby, our findings provide a promising therapeutic target and focus for clinical sepsis.

## Data Availability Statement

The original contributions presented in the study are included in the article/supplementary material. Further inquiries can be directed to the corresponding author.

## Ethics Statement

The animal study was reviewed and approved by Animal Management and Use Committee of Huazhong University of Science and Technology.

## Author Contributions

Design of the work: RQ, WY and YF; Data acquisition: RQ, CC, YZ and XZ; Data analysis: RQ, CC; Manuscript drafting: RQ, CC, YZ and XZ; Manuscript revision: CC, WY and YF. All authors contributed to the article and approved the submitted version.

## Funding

This study was funded by the National Natural Science Foundation of China [grant no. 82070572, 81570501, 81770554, 81772607, 81974383].

## Conflict of Interest

The authors declare that the research was conducted in the absence of any commercial or financial relationships that could be construed as a potential conflict of interest.
